# Supplementary material for: Super Enhancer-Regulated LINC00094 (SERLOC) Upregulates the Expression of MMP-1 and MMP-13 and Promotes Invasion of Cutaneous Squamous Cell Carcinoma
Source: Cancers (Basel). 2022 Aug 17;14(16):3980. doi: 10.3390/cancers14163980 (PMC9406669; doi:10.3390/cancers14163980)

Supplementary Material

Super enhancer regulated *LINC00094* (*SERLOC*) upregulates the expression of MMP-1 and MMP-13 and promotes invasion of cutaneous squamous cell carcinoma

Minna Piipponen, Pilvi Riihilä, Jaakko S. Knuutila, Markku Kallajoki, Veli-Matti Kähäri and Liisa Nissinen

Supplementary Figures S1-S5

Supplementary Tables S1 and S2

Original Western blots for Figure 5 and Supplementary Figures S3 and S4

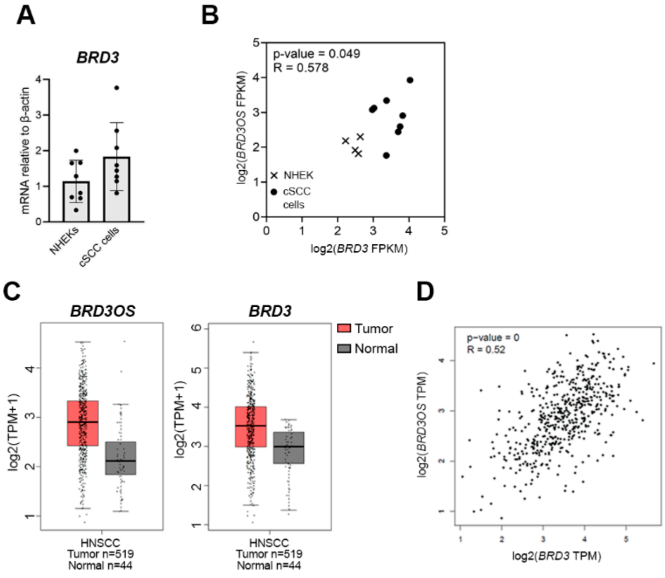

Supplementary Figure S1. Correlation of the expression of *BRD3OS (LINC00094)* and *BRD3* in cSCC cell lines and NHEKs and in head and neck SCCs (HNSCCs). A: *BRD3* expression was measured by qRT-PCR of normal human epidermal keratinocytes (NHEKs) (n=8) and cSCC cell lines (n=8). B: Correlation plot of *BRD3OS (LINC00094)* and *BRD3* expression measured by RNA-seq in cSCC cell lines (n=8) and NHEKs (n=4). Pearson correlation. C: *BRD3OS (LINC00094)* and *BRD3* mRNA expression data of HNSCC tumors and normal tissues from TCGA database. D: Correlation plot of *BRD3OS (LINC00094)* and *BRD3* expression in HNSCCs. Pearson correlation.

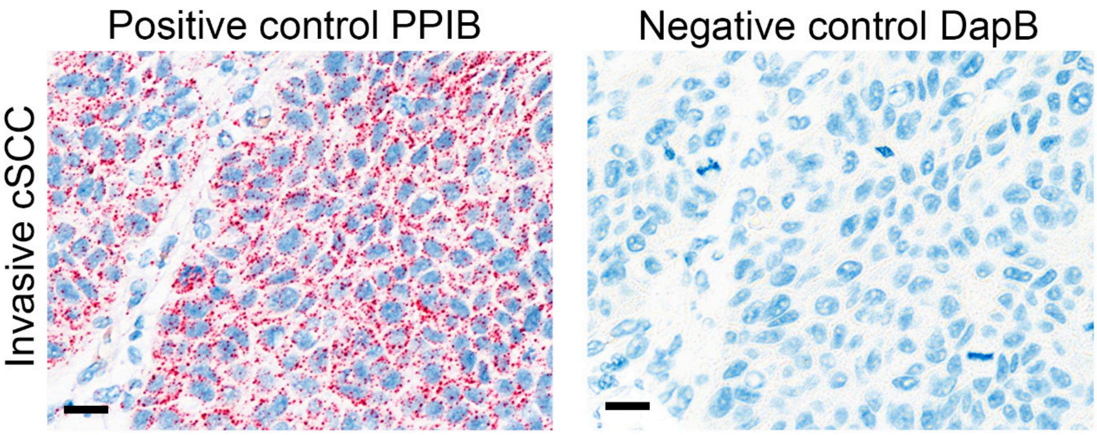

Supplementary Figure S2. Representative images of positive and negative controls of RNA *in situ* hybridization (RNA-ISH) in invasive cSCC tissue slide. Specific probes for human Cyclophilin B (PPIB) and bacterial 4-hydroxy-tetrahydrodipicolinate reductase (DapB) mRNAs were used as positive and negative controls for RNA-ISH, respectively. Scale bar = 20 µm.

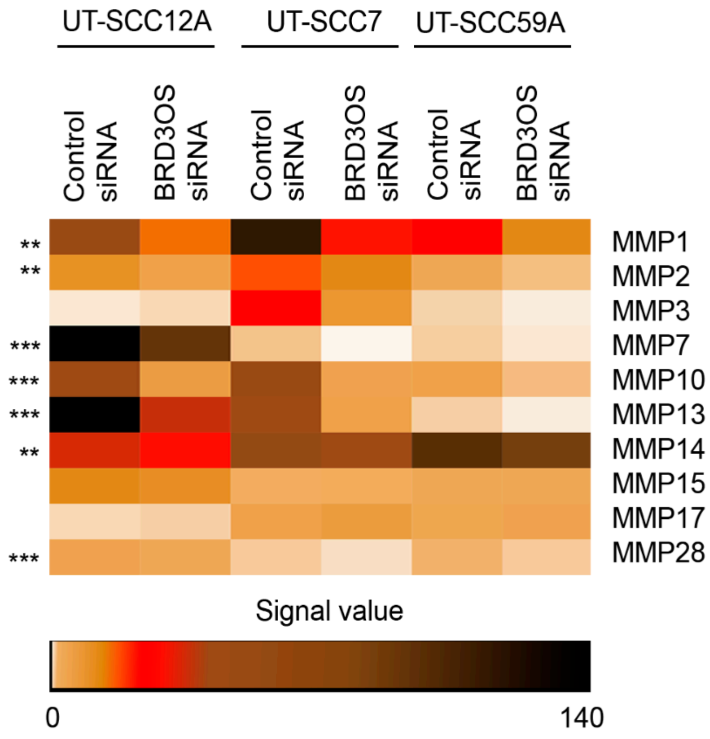

Supplementary Figure S3. Regulation of MMPs after *BRD3OS (LINC00094)* knockdown in cSCC cells. cSCC cell lines (UT-SCC12, UT-SCC59A and UT-SCC7) were transfected with negative control siRNA or *BRD3OS* siRNA_4 and after 3 days total RNA was isolated and RNA-seq was prepared. The expression of MMPs after *BRD3OS (LINC00094)* knockdown is shown as a heatmap. **p<0.01, ***p<0.001. R package Limma.

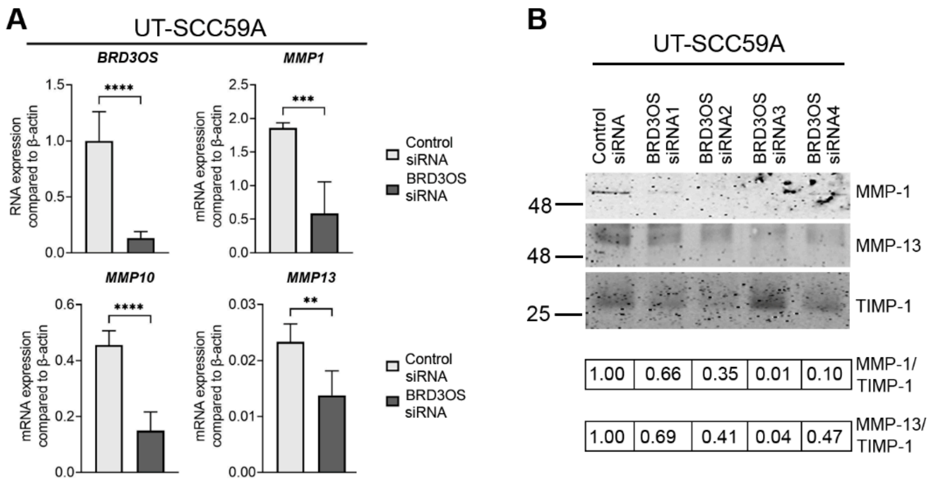

Supplementary Figure S4. MMP-1 and MMP-13 are downregulated in UT-SCC59A cells after *BRD3OS (LINC00094)* knockdown. A: *BRD3OS (LINC00094)*, *MMP1, MMP10 and MMP13* mRNA levels were determined by qPCR 72 h after *BRD3OS (LINC00094)* knockdown. Average of *BRD3OS (LINC00094)* knockdown using four different siRNAs targeting *BRD3OS (LINC00094)* is shown. B: Levels of MMP-1 and MMP-13 in conditioned media were determined by Western blotting 72 h after *BRD3OS (LINC00094)* knockdown. TIMP-1 was used as the loading control. Mean + SD is shown, **p<0.01, ***p<0.001, ****p<0.0001. Student's t-test.

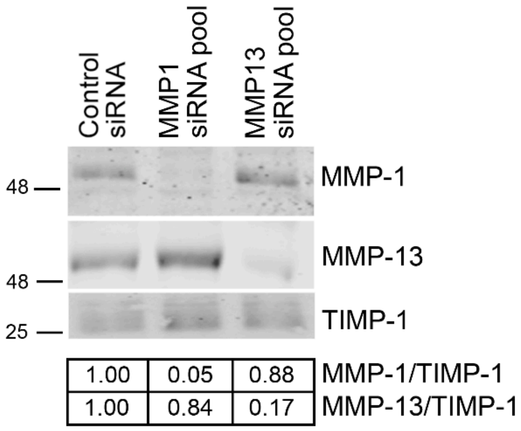

Supplementary Figure S5. Western blot analysis of MMP-1 and MMP-13 expression in cSCC cell culture medium 72 h after transfection of negative control and *MMP1* or *MMP13* siRNAs. TIMP-1 expression was used as loading control.

Supplementary Table S1. List of specific primers and probes for real-time quantitative PCR.

Gene/RNA	Sequence	
BRD3OS (LINC00094)	Forward	5'-CCG GCC CAC TTC ATG CCT T-3'
	Reverse	5'-CAA CCT GGA TGC TGT CAC TGT-3'
	Probe	5'-FAM-ACC TCT GAC TCG CAG CTC TCC CG-BHQ1-3'
MMP1	Forward	5′-AAGATGAAACGTGGACCAACAATT-3′
	Reverse	5′-CCAAGAGAATGGAAGAGTTC-3′
	Probe	5′-FAM-CAGAGAGTACAACTTACATCGTGTTGCGGCTC-TAMRA-3′
MMP10	Forward	5′-GGACCTGGGCTTTATGGAGATAT-3′
	Reverse	5′-CCCAGGGAGTGGCCAAGT-3′
	Probe	5′-FAM-CATCAGGCACCAATTTATTCCTCGTTGCT-TAMRA-3′
MMP13	Forward	5′-AAATTATGGAGGAGATGCCCATT-3′
	Reverse	5′-TCCTTGGAGTGGTCAAGACCTAA-3′
	Probe	5′-FAM-CTACAACTTGTTTCTTGTTGCTGCGCATGA-TAMRA-3′
ACTB	Forward	5′-TCACCCACACTGTGCCCATCTACGC-3′
	Reverse	5′-CAGCGGAACCGCTCATTGCCAATGG-3′
	Probe	5′-FAM-CAGCGGAACCGCTCATTGCCAATGG-BHQ1-3′
GAPDH	Forward	5'- ACC CAC TCC TCC ACC TTT GA -3'
	Reverse	5'- TTG CTG TAG CCA AAT TCG TTG T-3'
	Probe	5' -FAM-ACG ACC ACT TTG TCA AGC TCA TTT CCT GGT-BHQ1-3'

ACTB, ß-actin; *MMP1*, matrix metallopeptidase 1; *MMP10*, matrix metallopeptidase 10; *MMP13*, matrix metallopeptidase 13;

Supplementary Table S2. List of siRNAs used for silencing the genes indicated.

Target	siRNA name	Cataloque number	target sequence 5' --> 3'
BRD3OS (LINC00094)	BRD3OS siRNA_1	SI04931871	CAGGCTCTCCACCAGACTTAA
BRD3OS (LINC00094)	BRD3OS siRNA_2	SI04933089	AAGCATCATGGTAGCTTCCAA
BRD3OS (LINC00094)	BRD3OS siRNA_3	SI04933166	AACTGTGGTGTAAGAACCCAA
BRD3OS (LINC00094)	BRD3OS siRNA_4	SI04935686	ACCAGGAGCTTTGTCGCATTA
MMP1	MMP1 siRNA_12	SI05160141	ATGATGAATATAAACGATCTA
MMP1	MMP1 siRNA_7	SI03033772	AAGCTAACCTTTGATGCTATA
MMP1	MMP1 siRNA_6	SI03021802	TTCAGTGGTGATGTTCAGCTA
MMP1	MMP1 siRNA_1	SI00037492	AAGCGTGTGACAGTAAGCTAA
MMP13	MMP13 siRNA_8	SI03075947	CCCAACCGTATTGATGCTGCA
MMP13	MMP13 siRNA_7	SI03051013	ATGGATAAAGACTATCCGAGA
MMP13	MMP13 siRNA_6	SI03049277	ATGACTCATTCTGAAGTCGAA
MMP13	MMP13 siRNA_1	SI00037660	AACGAAATATCAAAGTCATTA

MMP1 matrix metallopeptidase 1; *MMP10*, matrix metallopeptidase 10; *MMP13*, matrix metallopeptidase 13;

Original Western blots for Figure 5B

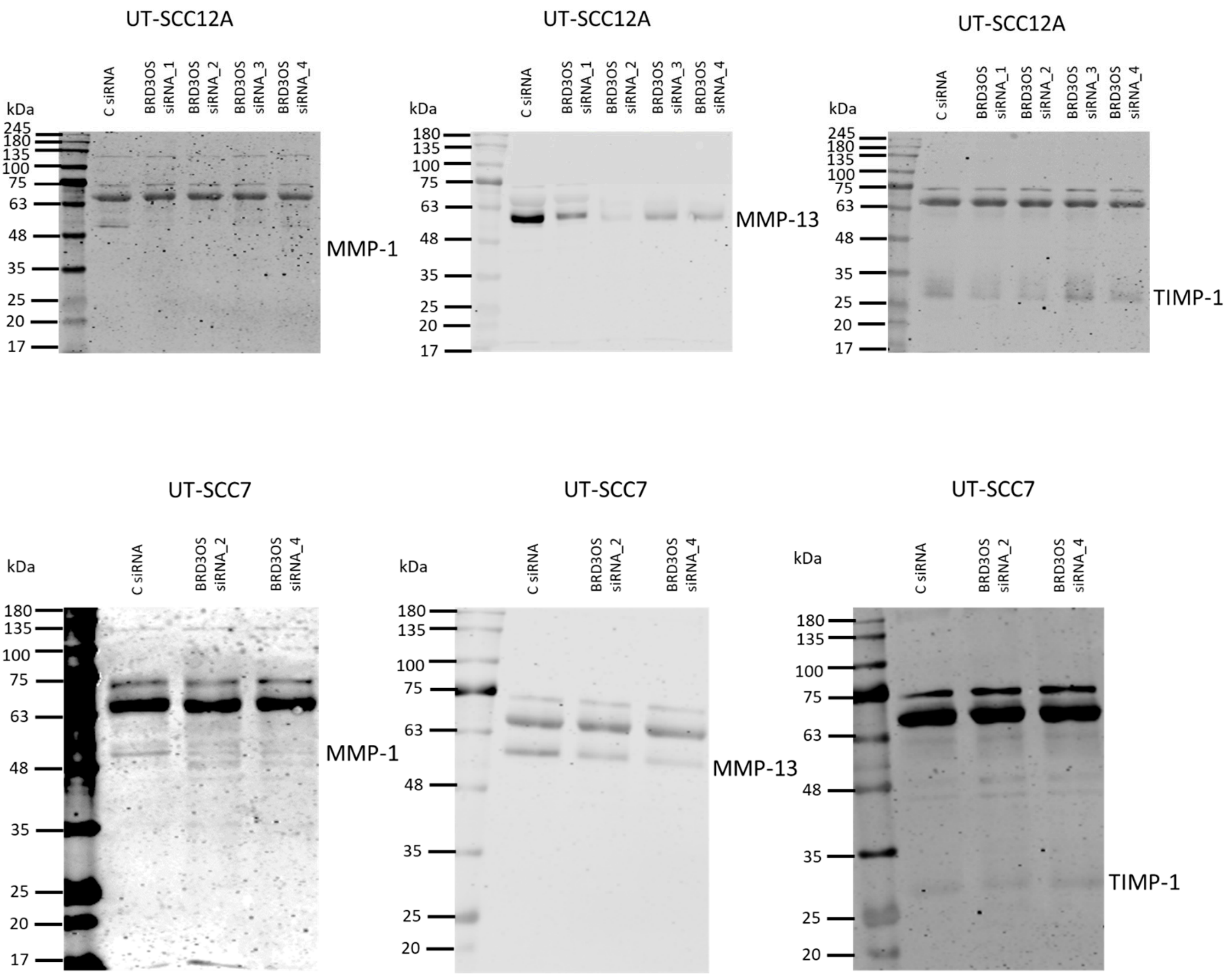

Original Western blots for Supplementary Figure S4

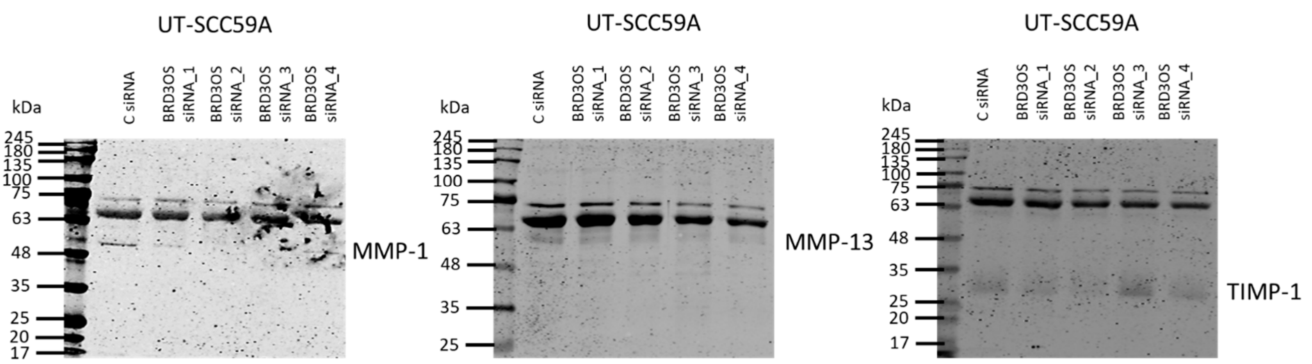

Original Western blots for Supplementary Figure S5

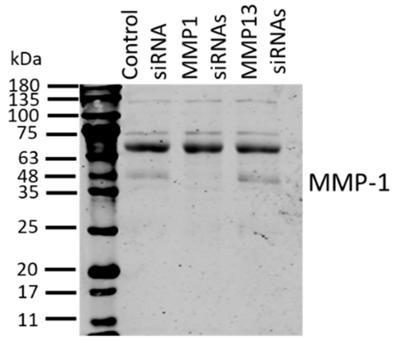

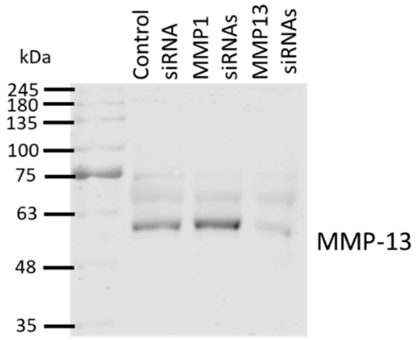

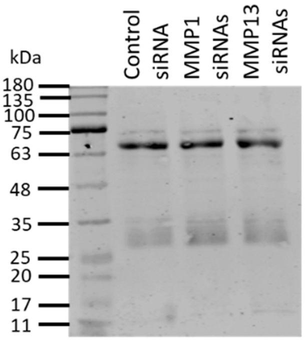

Supplement: Supplementary file 1 [file cancers-14-03980-s001.zip › cancers-1822133-supplementary.pdf]
